# Impact of COVID-19-adapted guidelines using different airway management strategies on resuscitation quality in out-of-hospital-cardiac-arrest – a randomised manikin study

**DOI:** 10.1186/s12873-023-00820-y

**Published:** 2023-05-15

**Authors:** Sean S. Scholz, Sissy Linder, Eugen Latka, Tobias Bartnick, Daniel Karla, Daniel Thaemel, Marlena Wolff, Odile Sauzet, Sebastian W. Rehberg, Karl-Christian Thies, Gerrit Jansen

**Affiliations:** 1 Department of Anaesthesiology, Intensive Care, Emergency Medicine, Transfusion Medicine, and Pain Therapy, University Hospital OWL, Protestant Hospital of the Bethel Foundation, University of Bielefeld, Burgsteig 13, 33617 Bielefeld, Germany; 2 Skillslab, Medical School East Westphalia-Lippe, Bielefeld University, Universitätsstraße 25, 33615 Bielefeld, Germany; 3 Department of Medical and Emergency Services, Study Institute Westfalen-Lippe, Remterweg 44, D-33617 Bielefeld, Germany; 4 Epidemiology and International Public Health, Bielefeld School of Public Health & Center for Statistics, Bielefeld University, Bielefeld, Germany; 5 Medical School, Bielefeld University, University Medical Center East Westphalia-Lippe, Universitätsstraße 25, 33615 Bielefeld, Germany; 6 University Department of Anaesthesiology, Intensive Care Medicine and Emergency Medicine, Johannes Wesling Klinikum Minden, Ruhr University Bochum, Hans-Nolte-Straße 1, 32429 Minden, Germany

**Keywords:** Aerosol liberation, CPR, Extraglottic airway, Supraglottic airway, Chest compression

## Abstract

**Background:**

Although airway management for paramedics has moved away from endotracheal intubation towards extraglottic airway devices in recent years, in the context of COVID-19, endotracheal intubation has seen a revival. Endotracheal intubation has been recommended again under the assumption that it provides better protection against aerosol liberation and infection risk for care providers than extraglottic airway devices accepting an increase in no-flow time and possibly worsen patient outcomes.

**Methods:**

In this manikin study paramedics performed advanced cardiac life support with non-shockable (Non-VF) and shockable rhythms (VF) in four settings: ERC guidelines 2021 (control), COVID-19-guidelines using videolaryngoscopic intubation (COVID-19-intubation), laryngeal mask (COVID-19-Laryngeal-Mask) or a modified laryngeal mask modified with a shower cap (COVID-19-showercap) to reduce aerosol liberation simulated by a fog machine. Primary endpoint was no-flow-time, secondary endpoints included data on airway management as well as the participants‘ subjective assessment of aerosol release using a Likert-scale (0 = no release–10 = maximum release) were collected and statistically compared. Continuous Data was presented as mean ± standard deviation. Interval-scaled Data were presented as median and Q1 and Q3.

**Results:**

A total of 120 resuscitation scenarios were completed. Compared to control (Non-VF:11 ± 3 s, VF:12 ± 3 s) application of COVID-19-adapted guidelines lead to prolonged no-flow times in all groups (COVID-19-Intubation: Non-VF:17 ± 11 s, VF:19 ± 5 s;p ≤ 0.001; COVID-19-laryngeal-mask: VF:15 ± 5 s,p ≤ 0.01; COVID-19-showercap: VF:15 ± 3 s,p ≤ 0.01). Compared to COVID-19-Intubation, the use of the laryngeal mask and its modification with a showercap both led to a reduction of no-flow-time(COVID-19-laryngeal-mask: Non-VF:p = 0.002;VF:p ≤ 0.001; COVID-19-Showercap: Non-VF:p ≤ 0.001;VF:p = 0.002) due to a reduced duration of intubation (COVID-19-Intubation: Non-VF:40 ± 19 s;VF:33 ± 17 s; both p ≤ 0.01 vs. control, COVID-19-Laryngeal-Mask (Non-VF:15 ± 7 s;VF:13 ± 5 s;p > 0.05) and COVID-19-Shower-cap (Non-VF:15 ± 5 s;VF:17 ± 5 s;p > 0.05). The participants rated aerosol liberation lowest in COVID-19-intubation (median:0;Q1:0,Q3:2;p < 0.001vs.COVID-19-laryngeal-mask and COVID-19-showercap) compared to COVID-19-shower-cap (median:3;Q1:1,Q3:3 p < 0.001vs.COVID-19-laryngeal-mask) or COVID-19-laryngeal-mask (median:9;Q1:6,Q3:8).

**Conclusions:**

COVID-19-adapted guidelines using videolaryngoscopic intubation lead to a prolongation of no-flow time. The use of a modified laryngeal mask with a shower cap seems to be a suitable compromise combining minimal impact on no-flowtime and reduced aerosol exposure for the involved providers.

**Supplementary Information:**

The online version contains supplementary material available at 10.1186/s12873-023-00820-y.

## Background

Based on experience with SARS-CoV-1, the resuscitation guidelines were adapted to mitigate the risk of infection for the rescuers, while accepting an increase in no-flow time and possibly worse patient outcomes [1–8]. Recent studies regarding out-of-hospital cardiac arrest (OHCA) demonstrated worse outcomes during the COVID-19 pandemic [9–16]. Potential explanations were lower survival rates caused by COVID-19, excessive utilization of health care capacities, prolonged response times due to donning of PPE, and the adaptations of resuscitation protocols recommending prolonged pauses in chest compressions for advanced airway management [1, 9–11, 17, 18]. Over the past years, prehospital airway management for paramedics has moved away from endotracheal intubation (ETI) towards supraglottic airway devices (SAD), skill acquisition and skill retention for ETI have proven difficult and there is evidence that SAD are non-inferior to ETI in OHCA [1,19.20,21]. In the context of COVID-19, however, ETI has made a comeback. It has been recommended when the condition is met that its complete seal of the trachea provides better protection against aerosol liberation and lowers infection risk for healthcare providers when compared to SAD or Bag-Mask-Ventilation during cardiopulmonary resuscitation (CPR) [1–3]. While studies have shown that the use of protective tents in the context of ETI led to a reduction in aerosol release, the effects of a protective film while using a SAD have not yet been investigated [22, 23]. Ideally, such protective film would allow the mouth-nose area to be sealed, leaving the neck, chest and extremities free for chest compressions, defibrillation and catheterisation. A modified SAD with a simple shower cap could therefore be suitable to elicit appropriate coverage. Due to the elastic band, it can be individually adjusted to the face of the wearer. The primary aim of the present study was to examine the impact of a laryngeal mask modified with a shower cap and other airway management strategies on no-flow-time and aerosol release against the background of the COVID-19 pandemic.

## Methods

**Fig. 1:**
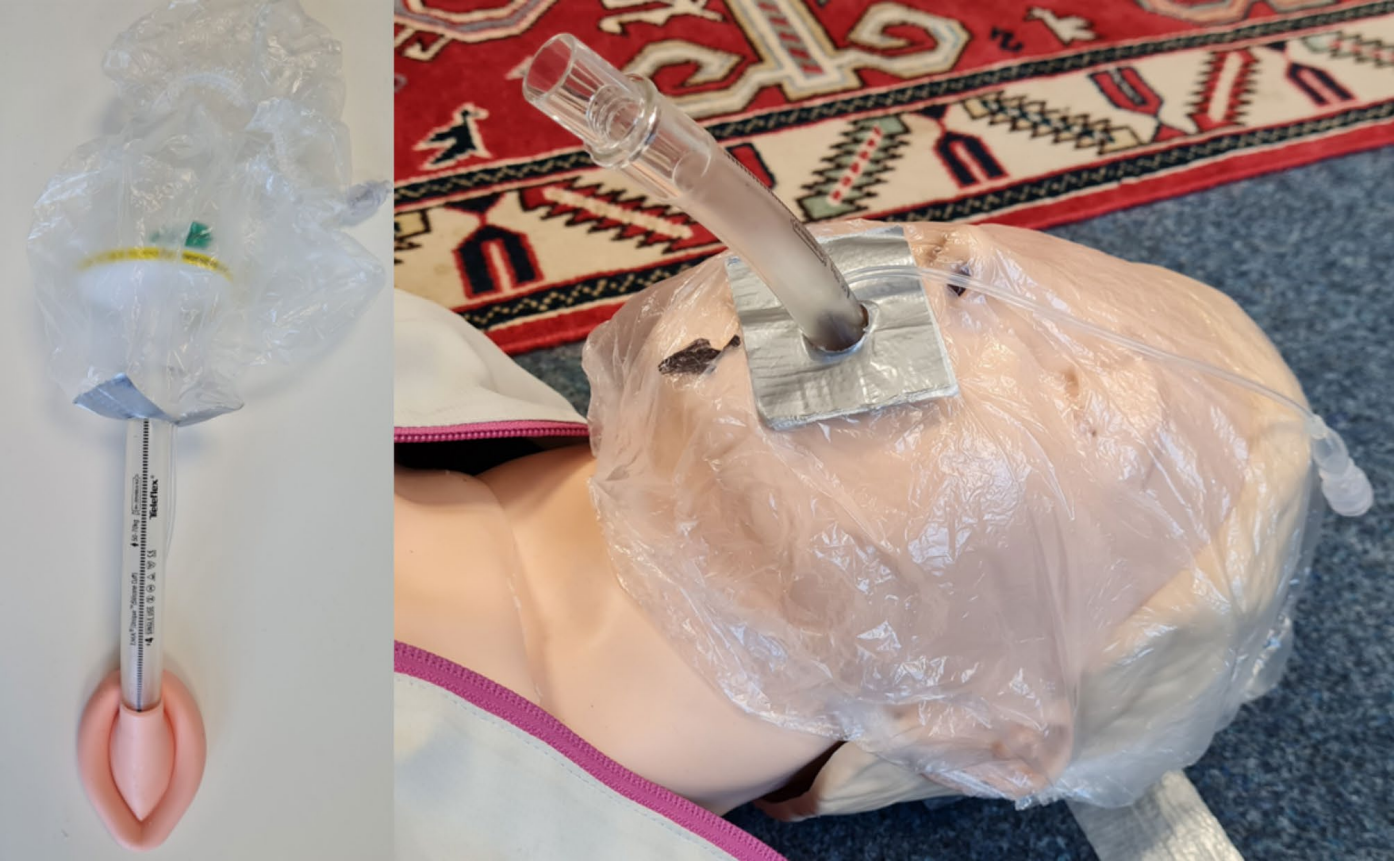
The laryngeal mask modified with a shower cap

Ethical approval for this study (Ethical Committee No 2021-414-f-S) was granted on July, 2th 2021 by the Ethical Committee of the University Hospital of Muenster, Muenster, Germany (Chairperson Prof W.E. Berdel). The study was performed at a training center for paramedics in Bielefeld, Germany in August 2021 (‘Studieninstitut für kommunale Verwaltung Westfalen-Lippe, Fachbereich Medizin und Rettungswesen’). This manuscript adheres to the applicable CONSORT guidelines. After providing written informed consent, 60 paramedics were randomly allocated into 30 fixed teams, simulating the crew of an ambulance, and asked to perform a basic-life-support (BLS) OHCA scenario according to the guidelines of the European Resuscitation Council (ERC) 2021 [24]. All paramedics were Advanced Life Support trained prior to the inclusion. Before the scenario, the participants were intensely trained using manakin simulation training in the ERC COVID-19-adapted guidelines. The training focused on modified procedures of basic- and advanced-cardiac-life-support (detection of cardiac arrest, covering the face with an oxygen mask before starting chest compressions, early use of a defibrillator) and three different methods of airway-management with the intention to minimize aerosol release and maximize provider protection [1–3]: Performing a videolaryngoscopic intubation (COVID-19-Intubation), placing a LMA (COVID-19-Laryngeal-Mask) and placing a modified LMA with a shower cap attached, which was placed over the patients face in order to catch aerosol leaking from there the airway during CPR (COVID-19-Shower-cap; See Fig. 1). Supplement 1 shows the modification of the algorithm used (see Supplement 1). Instructions for constructing the modified LMA are shown in Supplement 2.

**Fig. 2:**
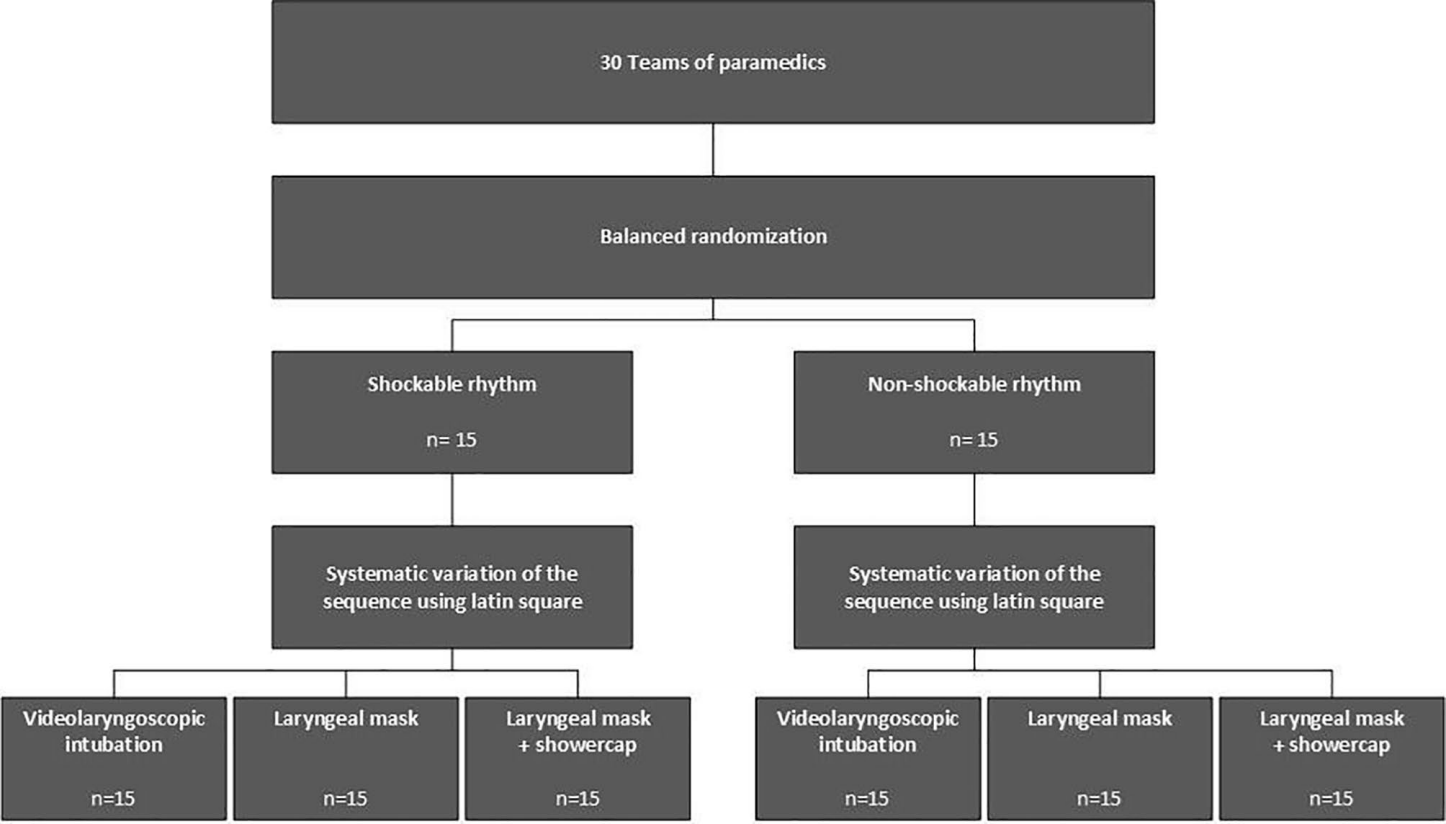
Flowchart of the group allocation process

Following the training, the previously formed teams were randomised via balanced randomization into two groups to investigate the effects on each initial rhythm: The first group completed the three scenarios of ERC COVID-19-adapted guidelines with a shockable, the second group with a non-shockable rhythm using each of the previously trained airway devices [2, 3]. To control for learning effects during the scenarios, the order of application of the different airway devices was systematically varied according to a Latin square. Figure 2 shows the group allocation process performed in the study.

The target for the participants was to achieve the highest possible resuscitation quality with regard to protection specificities due to the COVID-19 pandemic [1–3]. The time required for airway management (intervals to application of oxygen, first airway management attempt, successful intubation/placement of the LMA, duration of intubation/LMA placement attempts, number of intubation/LMA placement attempts, unsuccessful intubation/LMA placement attempts, false intubations/LMA placement and first ventilation) as well as indicators of resuscitation quality according to current ERC guidelines (no-flow-time = time without chest compressions, total number of compressions, depth of compressions, compression frequency, proportion of compressions with correct hand-position and sufficient compression depth, time to first rhythm analysis and defibrillation) were measured [2, 3, 24].

The Laerdal Resusci Anne QCPR manikin (Laerdal Medical GmbH, Lilienthalstr.5, 82,178 Puchheim, Germany, http://www.laerdal.com/) was used. The participants’ equipment consisted of a fully equipped emergency backpack (“jump bag”), a ventilator (Medumat Standard²; Weinmann Emergency Medical Technology GmbH + Co. KG, Frohbösestraße 12, D-22,525 Hamburg, Germany, https://www.weinmann-emergency.com) and a transportable monitor-defibrillator (corpuls3, GS Elektromedizinische Geräte G. Stemple GmbH, Hauswiesenstraße 26, D-86,916 Kaufering, https://corpuls.world/). For videolaryngoscopic intubation the i-view TM videolaryngoscope was used (Intersurgical GmbH, Siegburger Straße 39, D-53,757 Sankt Augustin, https://de.intersurgical.com/info/iview); for SAD the LMA Classic size four (Teleflex Medical GmbH, Welfenstrasse 19, D-70,736 Fellbach, Germany, https://teleflex.com/emea/de/index), inflated with 30ml of air to reach a cuff pressure of 60cmH2O, as measured by universal cuff pressure measuring device (VBM Medizintechnik GmbH, Einsteinstrasse 1, D-72,172 Sulza.N., www.vbm-medical.de) was used.

To visualize the simulated aerosol release, the breathing system of the Laerdal Resusci Anne QCPR manikin was modified using the ‚Look Tiny Cx fog machine‘ (Look Solutions GmbH & Co. KG, Bünteweg 33, DE-30,989 Gehrden, https://www.looksolutions.com/) (See Supplement 3).

Data were transmitted via WLAN to the SimPad PLUS (SimPad PLUS, Laerdal Medical 2016, www.laerdal.com) with SkillReporter (Session Viewer, Laerdal Medical 2016, www.laerdal.com), preinstalled on the tablet. The individual datasets were saved and evaluated using the debriefing software Session Viewer 6.2.6400 by Laerdal.

The duration of each scenario was limited to 5 min. During the COVID-19 scenarios, participants wore PPE as recommended in the COVID-19-adapted guidelines (FFP3 mask (equivalent to the US N95 standard), eye and face protection, long-sleeved gown, and gloves) [1, 2, 5]. Importantly, donning the equipment was not part of the scenarios to ensure that only the effects of the algorithm changes were investigated. Previous studies suggest a timeframe of one to five minutes for donning PPE [1, 2, 5, 19].

After completion of all scenarios, the participants were asked to anonymously provide information on their subjective assessment of the amount of aerosol release (0 = no release – 10 = maximum release) and the feeling of safety using the different airway devices (safe, rather safe, rather unsafe, unsafe), the assessment of the quality of the different resuscitation scenarios with regard to the shortest possible no-flow time (good quality, rather good quality, rather bad quality, bad quality) and optimal protection of the rescuer (good protection, rather good protection, rather bad protection, bad protection).

The primary outcome was no-flow time, defined as time without chest compressions. Secondary outcomes were the established quality indicators of resuscitation as mentioned above as well as the results of the participants survey on rescuer safety.

### Analysis

STATA version 16 (StataCorp. 2019. Stata Statistical Software: Release 16. College Station, TX: StataCorp LLC.) was used for statistical analyses. The sample size was calculated with a standardized tolerance limit of one standard deviation and no difference, 20 participants were required for a power = 0.8 with a significance level of 0.05. Mean difference between outcomes for the scenarios within the COVID-19 groups and compared to control group are adjusted for the correlation between measures obtained by the same team by using mixed models. We performed a non-inferiority analysis in which one scenario is said to be non-inferior to reference scenario if the lower limit of 95% confidence interval of the mean differences is less than half a standard deviation of the outcome for the reference scenario. The statistical evaluation of the provider survey was performed with the Friedman- and Wilcoxon–test. The significance level was set at P ≤ 0.05.

## Results

Thirty different teams of paramedics (female = 39%, male = 61%) completed a total of 120 cardiac arrest scenarios apportioned equally to the four study groups.

Table 1 shows the results of circulatory and respiratory interventions. Compared to the control group (non-VF:11 ± 3 s; VF:12 ± 3 s), no-flow time was prolonged in the COVID-19-intubation (non-VF:17 ± 11 s,p ≤ 0.01; VF:19 ± 5 s, p ≤ 0.001), COVID-19-laryngeal-mask (non-VF:11 ± 4 s; VF:15 ± 5 s) and COVID-19-laryngeal-mask and -shower-cap (non-VF:11 ± 4 s; VF:15 ± 3 s) (each VF:p ≤ 0.01) groups. The results of the parameters for chest compressions are shown in supplement 4. The results of the comparison of COVID-19 groups with ERC-2021 are presented in Table 2. Table 3 contains the results of the comparison within the COVID-19 groups. None of the groups were non-inferior when compared to the control group.

**Table 1:** Circulatory and respiratory interventions

Parameter	ERC 2021 (n = 30)		COVID-19-Intubation (n = 30)		COVID-19-laryngeal-mask (n = 30)		COVID-19-shower-cap (n = 30)	
	Non-VF (n = 15)	VF (n = 15)	Non-VF (n = 15)	VF (n = 15)	Non-VF (n = 15)	VF (n = 15)	Non-VF (n = 15)	VF (n = 15)
**No-flow time (sec)**	11 ± 3*	12 ± 3	17 ± 11 * ‡ $	19 ± 5 § † ***	11 ± 4	15 ± 5 *	11 ± 4	15 ± 3 *
**Detection of cardiac arrest (sec)**	23 ± 5	22 ± 5	19 ± 4 §	20 ± 4	19 ± 4 §	21 ± 4	18 ± 5 §	21 ± 4
**Time to first chest compression (sec)**	26 ± 6	24 ± 6	23 ± 4 *	26 ± 7 #	21 ± 4 §	26 ± 5	22 ± 5 §	26 ± 5
**Time to first rhythm analysis (sec)**	58 ± 24	52 ± 12	50 ± 13 #	53 ± 12	50 ± 12 #	52 ± 10	46 ± 10 *	49 ± 8
**Time to second rhythm analysis (sec)**	193 ± 28	197 ± 16	186 ± 16 ***	192 ± 18	184 ± 12	192 ± 12	117 ± 10 *	194 ± 27
**Time to oxygen supply (sec)**	129 ± 20	112 ± 28	18 ± 4 §	23 ± 7 §	18 ± 4 §	23 ± 4 §	17 ± 5 §	23 ± 6 §
**Time to first ventilation (sec)**	129 ± 20	114 ± 26	152 ± 33 * † $	144 ± 39 § † $	109 ± 22 *	116 ± 15	112 ± 21 #	118 ± 22
**Time to first intubation attempt (sec)**	92 ± 40	86 ± 19	95 ± 20	98 ± 22 *	87 ± 17	97 ± 17 *	87 ± 15	94 ± 18
**Time to successful intubation (sec)**	113 ± 42	109 ± 17	97 ± 82	137 ± 38 § † $	99 ± 20	110 ± 16	96 ± 32	111 ± 17
**Duration of intubation (sec)**	21 ± 9	19 ± 9	40 ± 19 § † $	33 ± 17 § † $	15 ± 7	13 ± 5	15 ± 5	17 ± 5
**Total number of chest compressions (∑)**	449 ± 33	438 ± 35	402 ± 82 * † $	380 ± 44 § ** $	460 ± 38	407 ± 40 *	465 ± 38	416 ± 37 #
**Overall Resuscitation Quality (%)**	48 ± 21	45 ± 20	26 ± 17 § ‡	28 ± 17 * **	40 ± 13	42 ± 16	31 ± 13	38 ± 19

**Legend**: * p ≤ 0.01vs. Control Group; § p ≤ 0.001 vs. Control Group; # p ≤ 0.05 vs. Control Group

† p ≤ 0.001 vs. COVID-19-Intubation; ‡ p = 0.002 vs. COVID-19-Intubation, ** p ≤ 0.01 vs. COVID-19-Intubation

$ p ≤ 0.001 vs. COVID-19-shower-cap *** p = 0.002 vs. COVID-19-shower-cap

COVID-19 = Corona-Virus-Disease 2019; ERC = European Resuscitation Council; mg = milligram; mm = millimeters; min = minute; Non-VF = Non-shockable initial rhythm; sec = second, VF = Shockable initial rhythm

data are presented as mean ± standard deviation

**Table 2:** Results of the non-inferiority trial COVID-19-groups vs. ERC-2021

Parameter	COVID-19–Intubation (mean difference [95%CI])		COVID-19-laryngeal-mask (mean difference [95%CI])		COVID-19-shower-cap (mean difference [95%CI])	
	Non-VF	VF	Non-VF	VF	Non-VF	VF
**No-flow time (sec)**	5.9 [1.8–10.0] *	7.5 [5.1–9.9] §	-0.4 [-4.6–3.8]	3.5 [1.1–5.9] *	-0.8 [-5.0–3.4]	3.7 [1.3–6.1 ] *
**Detection of cardiac arrest (sec)**	-4.5 [-6.9 - -2.1] §	-0.5 [-2.3–1.2]	-4.6 [-7 - -2.1] §	-0.1 [-1.9 -1.7]	-5.7 [-8.1 - -3.3] §	-0.3 [-2.1–1.4]
**Time to oxygen supply(sec)**	-111.9 [-119.5 - -104.4] §	− 92.0 [-99.6 - -84.5] §	-111.5 [-119.1 - -103.8] §	-92.1 [-99.6 - − 84.5] §	-112.4 [-120.0 - -104.7] §	-92.3 [-99.8 - -84.7] §
**Time to first chest compression (sec)**	-3.8 [-6.4 - -1.2] *	3.2 [0.2–6.1] #	-4.6 [-7.3 - -2.0] $	2.9 [-0.1–5.9]	− 4.7 [-7.3 - -2.1] §	2.8 [-0.1–5.8]
**Time to first ventilation (sec)**	21.6 [5.1–38.2] *	23.2 [9.8–36.6] §	-21.5 [-38.3 - -4.6] *	-5.6 [-19.0–7.8]	-18.0 [-34.9 - -1.2] #	− 2.7 [-16.1–10.7]
**Time to first rhythm analysis (sec)**	-6.2 [-12.9–0.5]	1.4 [-4.6–7.6]	-6.8 [-13.5 - -0.02] #	-0.2 [-6.2–5.9]	-10.6 [-17.3 - -3.8] *	-2.5 [-8.6–3.6]
**Time to second rhythm analysis (sec)**	-4.35 [-14.1–5.4]	-5.3 [-17.4–6.7]	-7.6 [-17.5–2.1]	-5.4 [-17.5–6.6]	-14.9 [-24.9 - -4.8] *	-4.2 [-16.3–7.9]
**Time to first intubation attempt (sec)**	2.8 [-14.5–20.0]	13.3 [3.8–22.9] *	-5.9 [-23.5–11.6]	12.1 [2.6–21.7] *	-5.5 [-23.1–12.0]	8.9 [-0.7–18.4]
**Time to successful intubation (sec)**	-16.3 [-51.2–18.6]	21.0 [13.7–40.3] §	-13.7 [-49.2–21.8]	0.2 [-13.1–13.5]	-17.2 [-52.8–18.3]	0.5 [-12.8–13.8]
**Duration of intubation (sec)**	18.2 [10.0-26.5] §	14.1 [7.5–20.8] §	-6.6 [-15.0–1.7]	-5.9 [-12.5–0.8]	-5.9 [-14.2–2.5]	− 2.2 [-8.9–4.4]
**Total number of chest compressions**	-43.0 [-74.3- -11.6] *	− 61.7 [-83.7 - -39.7] §	15.3 [-16.4–47.1]	-34.9 [-56.9 - -12.9] *	20.5 [-11.2–52.3]	-25.7 [-47.7 - -3.7] #
**Overall Resuscitation Quality (%)**	-23.7 [-34.3 - -13.1] §	-17.8 [-28.9 - -6.7] *	-9.7 [-20.4–1.09]	-3.5 [-14.6–7.6]	-18.3 [-29.0–7.5]	− 8.2 [-19.2–2.9]

**Legend:** * p ≤ 0.01vs. Control Group; § p ≤ 0.001 vs. Control Group; # p ≤ 0.05 vs. Control Group

COVID-19 = Corona-Virus-Disease 2019; Non-VF = Non-shockable initial rhythm; sec = second, VF = Shockable initial rhythm; 95%CI = 95% confidence intervall

**Table 3:** Results of the non-inferiority trial within COVID-19-groups

	COVID-19-laryngeal-mask vs. COVID-19-Intubation (mean difference [95%CI])		COVID-19-Intubation vs. COVID-19-shower-cap (mean difference [95%CI])		COVID-19-laryngeal-mask vs. COVID-19-shower-cap (mean difference [95%CI])	
	Non-VF	VF	Non-VF	VF	Non-VF	VF
**No-flow time (sec)**	-6.3 [-10.3 – -2.2] #	-4 [-6.4 – -1.6] §	6.7 [2.7–10.8] §	3.9 [1.5–6.3] #	0.4 [-3.7–4.5]	-0.1 [-2.5–2.3]
**Time to first ventilation (sec)**	-43.1 [-59.6 – -26.6] §	-28.9 [-41.4 – -16.3] §	39.7 [23.2–56.2] §	25.9 [13.4–38.5] §	-3.4 [-20.2–13.3]	-2.9 [-15.5–9.6]
**Time to second rhythm analysis (sec)**	-3.3 [-11.9–5.3]	-0.1 [-12.4–12.1]	10.5 [1.7–19.3] *	-1.1 [-13.4–11.1]	7.2 [-1.6–16.1]	-1.3 [-13.5–11.0]
**Time to successful intubation (sec)**	2.6 [-32.9–38.1]	-26.8 [-39.9 – -13.7] §	1.0 [-34.52–36.5]	26.5 [13.5–39.6] § 00	3.6 [-32.6–39.7]	-0.3 [-13.3–12.8]
**Duration of intubation (sec)**	-24.9 [-33.3–16.5] §	-20.0 [-27.0 – -13.0] §	24.1 [15.8–32.5] §	16.3 [9.3–23.3] §	-0.8 [-9.3–7.7]	-3.7 [-10.7–3.3]
**Total number of chest compressions (sec)**	58.3 [28.6–88.0] §	26.8 [5.0–48.6] *	-63.5 [-93.2 - -33.8] §	-36.0 [-57.8 - -14.2] §	-5.2 [-35.3–24.8]	-9.2 [-31.0–12.6]
**Overall Resuscitation Quality (%)**	14.0 [3.9–24.2] *	14.3 [3.2–25.5] *	-5.4 [-15.6–4.8]	-9.7 [-20.8–1.5]	8.6 [-1.6–18.9]	4.7 [-6.5–15.8]

**Legend:** § p ≤ 0.001; # p = 0.002; * p ≤ 0.01

COVID-19 = Corona-Virus-Disease 2019; Non-VF = Non-shockable initial rhythm; sec = second, VF = Shockable initial rhythm; 95%CI = 95% confidence intervall

Participants ranked aerosol release higher when using the LMA (mean:7.1 ± 2.0; median:9;Q1:6;Q3:8) compared to the LMA with shower cap (mean:2.4 ± 1.5; median:3, Q1:1,Q3:3) and intubation (1.1 ± 1.4, median:0,Q1:1,Q3 3) (each p < 0.001 vs. laryngeal mask). Using the LMA with shower cap was subjectively worse than ETI (p < 0.001). Participants felt safer (p < 0.001 each) using both the LMA with shower cap and ETI compared to the LMA, but not when comparing the LMA with shower cap with endotracheal intubation (p > 0.05). Within the COVID-19 groups, participants reported significantly improved resuscitation quality and shorter no-flow time for the COVID-19-Laryngeal-mask group when compared to the COVID-19-intubation group (p < 0.001), but more aerosol exposure than for COVID-19-intubation (p < 0.001). The results of the survey of the participants regarding the different algorithms are shown in Table 4. Supplements 5–8 show the aerosol release of the different airway devices.

**Table 4:** Results of the survey

	**Median (Q1; Q3)**
**How do you evaluate the aerosol release for the different respiratory devices?** **(No release = 0; Maximum release = 10)**	
· Laryngeal Mask	9 (6; 8) * +
· Laryngeal Mask + Shower cap	3 (1; 3) + §
· Intubation	0 (0; 2) * §
**How do you evaluate the Rescuers Safety for the different respiratory devices?** **(1 = Safe; 2 = rather safe; 3 = rather unsafe; 4 = unsafe)**	
· Laryngeal Mask	3 (2; 4) * +
· Laryngeal Mask + Shower cap	2 (1; 2) §
· Intubation	1 (1; 2) §
**How do you evaluate the Resuscitation Quality for the different algorithms performed?** **(1 = efficient; 2 = rather efficient; 3 = rather inefficient; 4 = inefficient)**	
· *ERC 2021*	1 (1; 1) § * +
· *COVID-19*	
· Laryngeal Mask	2 (1; 2) % $
· Laryngeal Mask + Shower cap	2 (2; 2) %
· Intubation	2 (2; 3) % §
**How do you evaluate the minimization of no-flow-time for the different algorithms performed?** **(1 = efficient; 2 = rather efficient; 3 = rather inefficient; 4 = inefficient)**	
· *ERC 2021*	1 (1; 1,25) § * +
· *COVID-19*	
· Laryngeal Mask	2 (1; 2) % $
· Laryngeal Mask + Shower cap	2 (1,5; 2) %
· Intubation	2 (2; 3) % ß
**How do you evaluate the optimal Rescuers Safety for the different respiratory devices?** **(1 = Safe; 2 = rather safe; 3 = rather unsafe; 4 = unsafe)**	
· *ERC 2021*	3 (2; 3)* +
· *COVID-19*	
· Laryngeal Mask	3 (2; 3) * +
· Laryngeal Mask + Shower cap	2 (1; 2) % §
· Intubation	1 (1; 2) % §

**Legend:** *P < 0.001 vs. Laryngeal-Mask + Shower cap

+ p < 0.001 vs. Intubation

§ p < 0.001 vs. Laryngeal-Mask

% p < 0.001 vs. ERC 2021

$ p = 0.01 vs. Intubation

ß = p = 0.01 vs. COVID-19-Laryngeal-Mask

ERC = European Resuscitation Council; COVID-19 = Coronavirus-Disease 2019; Q1 = First Quartile, Q3: Third Quartile

## Discussion

The present paper compares no-flow time and established quality indicators of resuscitation as well as aerosol release using COVID-19 resuscitation guidelines including three different airway management strategies in accordance with the ERC-2021 using a simulation model [1–3, 24]. Compared to ERC-2021, the COVID-19-adapted guidelines using ETI led to a prolongation of the no-flow time when compared to SAD. The use of modified laryngeal mask with a shower cap had minimal impact on no-flow time and markedly reduced aerosol exposure for the involved providers.

Over the last decade, the impact of different airway management strategies on outcomes following OHCA has been investigated in various studies [19, 20]. Although prolonged intubation attempts in particular were associated with a prolongation of the no-flow time, potentially leading to a delay in the rate of ROSC with poorer outcome [19, 20, 22, 24], ETI was explicitly recommended in the guidelines for COVID-19 resuscitation as the gold standard for reducing aerosol release in view of the idea of optimal protection of the rescuers [1, 2]. The present study supports previous data showing a prolongation of no-flow time during resuscitation even with the use of videolaryngoscopy and can thus at least partially explain the worsening of patients´ outcome at the time of the COVID-19 pandemic indicating that these need to be optimized in the light of future pandemics [1, 3, 9, 10, 16].

Since the insertion of SAD is easy to both learn and put into practice for non-medical emergency service personnel, their use is widespread in Europe. Due to the relevant risk of release of infectious aerosols, airway interventions in patients with COVID-19 are associated with a significant risk of infection. Especially during face mask and supraglottic airway ventilation, intubation, extubation, and cardiopulmonary resuscitation, an increased aerosolization could be observed [25–28]. Nevertheless, in COVID-19 resuscitation, the use of SAD by rescuers not experienced in ETI was recommended knowing well the higher risk of aerosol release [1, 3]. To minimise virus transmission, various studies investigated different barrier systems, such as intubation tents, bag barrier drape systems etc., showing that containment systems can reduce the spread of infectious respiratory secretions during simulated coughing or extubation [26–28]. Despite these advantages, these systems had disadvantages in resuscitation situations, such as the time required to set up the device for patient use, covering the airways as quickly and easily as possible, easy access to the airway device, containment of aerosolization, difficult access to the patient to place defibrillation electrodes or catheters, e.g. puncture of the internal jugular vein, or problems during patient transport, as not every intubation tent can be easily attached to an ambulance stretcher [26–28]. The present pilot study suggests that the use of a modified LMA with a shower cap may be a viable alternative for airway management in the context of a pandemic with droplet and/or aerosol transmissible pathogens [3]: Firstly, deteriorations regarding no-flow time and total number of chest compressions were only observed in the VF group, making the COVID-19-laryngeal-mask- and COVID-19-shower-cap-group the best performing section compared to the other study groups. Secondly, participants rated aerosol release as lower and rescuer safety as higher compared to the LMA without shower cap. Thirdly, the production of an appropriately modified airway is simple, inexpensive and would therefore be easy to implement even in low-income countries where vaccination strategies may not have been implemented across the board or in anti-vaccine countries. Fourthly, such a modified LMA would allow the mouth-nose area to be sealed, leaving the neck, chest and extremities free for chest compressions, defibrillation and catheterization. Finally, the relative ease of use of SAD has been curbed by concerns regarding an incomplete seal of the airways causing an increased risk of liberation of infectious aerosols. This is especially so in dynamic resuscitation scenarios with risk of dislocation of the airway device and when the cuff pressure was limited to 60 cmH20 as in the present study [3, 28–30]: The use of a modified LMA with a shower cap seems to be the optimal compromise between helper safety on the one hand and optimal resuscitation quality on the other [2, 30].

SAD manufacturers should consider incorporating the shower cap concept into their SAD and prospective studies should evaluate this concept with regard to complications such as obstructing access to the airway in situations such as vomiting, an additional risk be a unnoticed tear in the shower cap, which may lead to a false sense of safety, different head-shapes, patients with a large beard or additional wasted plastic for times when an SAD is placed but CPR is not being performed. In addition, an SAD modified with a shower cap could represent a further building block not only in the context of prehospital resuscitation or difficult airway protection but also for anaesthesia.

### Limitations

As the influence of the different airway devices on key performance indicators of resuscitation was investigated, the application of the PPE was not part of the study, but obviously leads to a further prolongation of no-flow time [1, 17]. Performing resuscitation under observation during the study may have influenced the participants (Hawthorne- effect). Furthermore, manikin-based investigations provide only a limited reproduction of reality e.g. the patients’ anatomy when inserting a SAD and patients` outcomes cannot be evaluated. The present study investigated aerosol liberation when a LMA classic is employed. Different kinds of SAD such as the laryngeal tube may have different leak pressures. However, it can be assumed that the use of a shower cap will lead to a reduction in aerosol exposure with other SAD also. Further studies are necessary to evaluate the influence of a shower cap in combination with different SAD. In addition, in the present study, simulation of aerosol liberation was simulated by means of a fog machine. Aerosol dispersion may not fully reflect reality. Quantity of aerosol release could not be measured objectively. Evaluation on the basis of the participants assessment has limitations. Currently, there are other methods to measure the aerosol release objectively, such as digital analysis of video recordings or optical particle sizer. Nevertheless, with regard to the heterogenous OHCA patients, a simulation model may provide standardized and valuable insights on optimum airway management in OHCA in the light of the COVID-19 pandemic. As a result, the observed effects may be of significance in view of the current situation with the spread of the COVID-19 pandemic and the expected deterioration in the quality of resuscitation because there are only limited possibilities for the urgently required clinical evaluation of the corresponding guideline changes. In addition, results for the COVID-19-adapted guidelines may improve following more intensified training of the providers under adequate hygiene concepts. Possibly non-inferiority could not be proven due to an insufficient number of participants. However, no-flow time did not differ between the LMA and shower cap groups, whereas aerosol release was rated lower so that an increase in the number of cases could prove non-inferiority.

## Conclusions

The present study shows that the COVID-19-adapted guidelines using ETI led to a prolongation of the no-flow time, which markedly worsened the overall quality of resuscitation. These effects can be attenuated using an SAD. Although the influence of airway management on the outcome of resuscitation and the transmission of Sars-CoV-2 is critically discussed, the use of a modified LMA with a shower cap seems to be suitable to ensure optimal resuscitation quality and to reduced aerosol exposure at the same time.

## Electronic supplementary material

Below is the link to the electronic supplementary material.

Supplementary Material 1

Supplementary Material 2

Supplementary Material 3

Supplementary Material 4

Supplementary Material 5

Supplementary Material 6

Supplementary Material 7

Supplementary Material 8

Supplementary Material 9

## Data Availability

The datasets used and/or analysed during the current study are available from the corresponding author on reasonable request.

## Abbreviations

BLS: Basic Life-Support
CPR: Cardiopulmonary Resuscitation
COVID-19: Corona-Virus-Disease 2019
ETI: Endotracheal Intubation
ERC: European Resuscitation Council 2021
EGA: Extraglottic Airway Devices
FFP3: Filtering Face Piece 3
LMA: Laryngeal Mask Airway
OHCA: Out-of-Hospital Cardiac Arrest
PPE: Personal Protective Equipment
SARS-CoV-1: Severe Acute Respiratory Syndrome Corona-Virus 1
US: United States
VF: Ventricular Fibrillation
