# Supplementary material for: Impact of COVID-19-adapted guidelines using different airway management strategies on resuscitation quality in out-of-hospital-cardiac-arrest – a randomised manikin study
Source: BMC Emerg Med. 2023 May 15;23:48. doi: 10.1186/s12873-023-00820-y (PMC10184619; doi:10.1186/s12873-023-00820-y)
Supplement: Supplementary file 1 — Supplementary Material 1 [file 12873_2023_820_MOESM1_ESM.docx]

**Supplemental Digital Content**

**Supplement 1:** The modified COVID-19-Algorithm

**Legend:** SAD = supraglottic airway; FIO2 = Inspiratory Fraction of Oxygen; Max = Maximum; Min = Minimum; MV = Minute Volume, PEA = Pulseless Electric Activity; RR = Respiratory Rate; VT = Tidal Volume; VF = Ventricular Fibrillation

**Supplement 2:** Instructions for making the laryngeal mask modified with a shower cap.

**Supplement 3:** Modified Laerdal manikin

**Supplement 4:** Quality parameters of chest compressions

**Supplement 5:** Aerosol release without airway device

**Supplement 6:** Aerosol release when using the Laryngeal Mask

**Supplement 7:** Aerosol release when using the Laryngeal Mask and Shower cap

**Supplement 8:** Aerosol release when using endotracheal intubation
