# Supplementary material for: Impact of COVID-19-adapted guidelines using different airway management strategies on resuscitation quality in out-of-hospital-cardiac-arrest – a randomised manikin study
Source: BMC Emerg Med. 2023 May 15;23:48. doi: 10.1186/s12873-023-00820-y (PMC10184619; doi:10.1186/s12873-023-00820-y)
Supplement: Supplementary file 2 — Supplementary Material 2 [file 12873_2023_820_MOESM2_ESM.pdf]

Supplement 1: The modified COVID-19-Algorithm

Suspected or confirmed Covid-19: Don airborne-precaution PPE

**Head position**	**Resuscitation**<br>Uncover the patient	**Lateral position**
Cover the victim´s nose and mouth with an oxygen		Unresponsive and not breathingnormally?
		Asses breathing visually; do not open the airway or approach the victim´s nose & mouth
**Prepare defibrillator**<br>Switch on. Attach the defibrillator electrodes. Charge defibrillator. if available: Prepare feedback system.		Chest compressions<br>100 - 120 / min.<br>Compression depths<br>5 - 6 cm
		Give continuous chest compressions<br>Minimise interruptions

First Evaluation of the heart rhythm
VT / VF: Defibrillation
Asystolia / PEA / ejectable rhythm: No Defibrillation; Discharge Defibrillator

Insert Airway<br>Start Ventilation<br>Use Capnographia<br>Charge defibrillator	If possible, continuous chest compression with minimal pauses<br><br>Ventilation:<br>RR: 10/min.<br>MV: 6l<br>FIO_2: 1.0<br>mechanical ventilation:<br>Pmax: Note max. airway pressure limits of the EGA<br>in case of leakage during Chest compressions -> 30:2	If available attach Feedback system<br>Chest compressions<br>100 - 120 / min.<br>Compression depths<br>5 - 6 cm

Second Evaluation of the heart rhythm; Change provider positions
VT / VF: Defibrillation
Asystolia / PEA / ejectable rhythm: No Defibrillation; Discharge Defibrillator

Charge Defibrillator	Re-Asses Airway and Breathing	Chest compressions<br>100 - 120 / min.<br>Compression depths<br>5 - 6 cm

Third Evaluation of the heart rhythm; Change provider positions
VT / VF: Defibrillation
Asystolia / PEA / ejectable rhythm: No Defibrillation; Discharge Defibrillator

Continue with resuscitation

Legend:
SAD = supraglottic airway; FIO2 = Inspiratory Fraction of Oxygen; Max = Maximum
Min = Minimum; MV = Minute Volume, PEA = Pulseless Electric Activity; RR = Respiratory Rate; VT = Tidal Volume; VF = Ventricular Fibrillation
