# Supplementary figures and images for: Impact of COVID-19-adapted guidelines using different airway management strategies on resuscitation quality in out-of-hospital-cardiac-arrest – a randomised manikin study
Source: BMC Emerg Med. 2023 May 15;23:48. doi: 10.1186/s12873-023-00820-y (PMC10184619; doi:10.1186/s12873-023-00820-y)

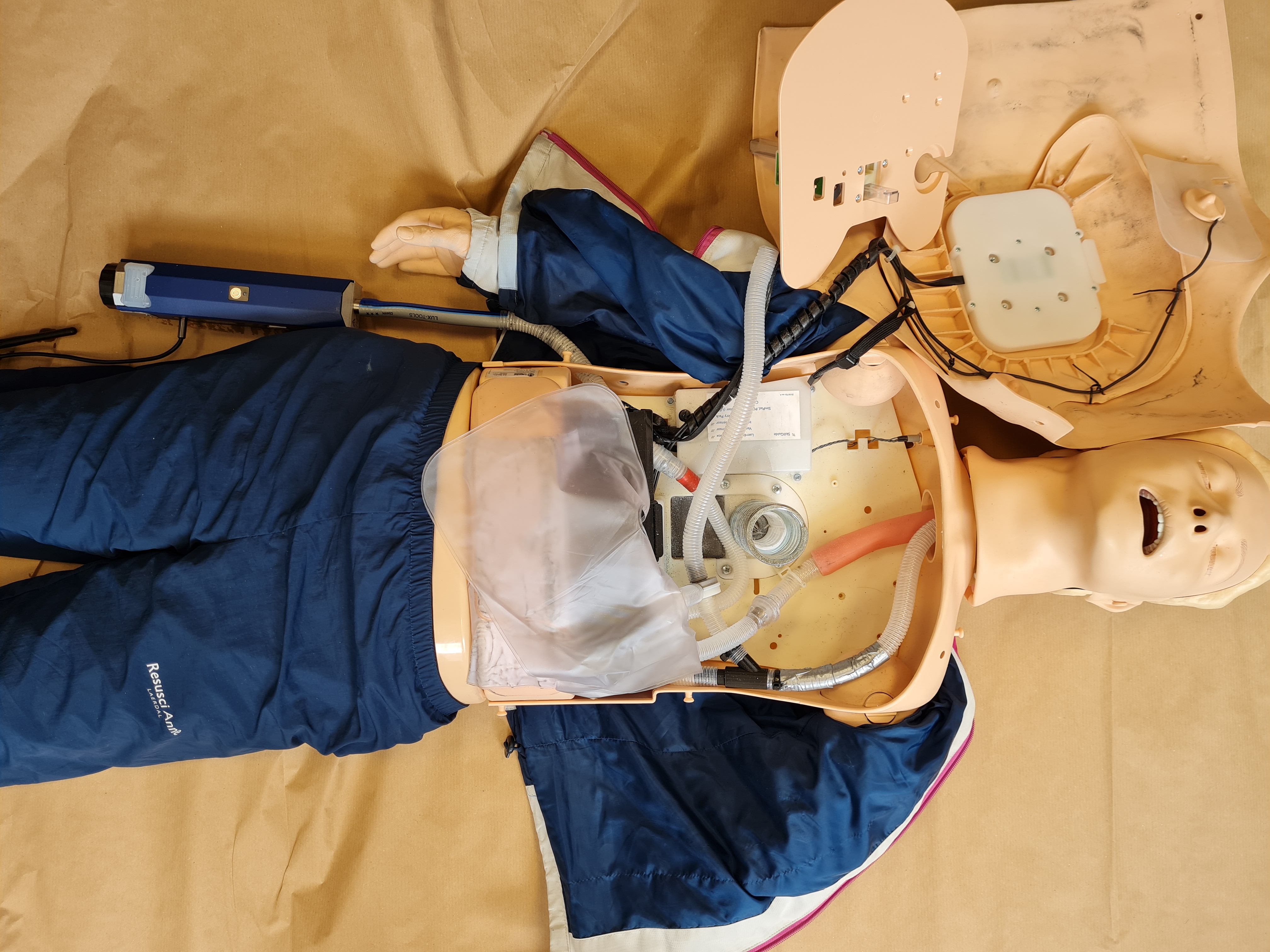

Supplement: Supplementary file 4 — Supplementary Material 4 [file 12873_2023_820_MOESM4_ESM.jpg]
