# Supplementary material for: Impact of COVID-19-adapted guidelines using different airway management strategies on resuscitation quality in out-of-hospital-cardiac-arrest – a randomised manikin study
Source: BMC Emerg Med. 2023 May 15;23:48. doi: 10.1186/s12873-023-00820-y (PMC10184619; doi:10.1186/s12873-023-00820-y)
Supplement: Supplementary file 5 — Supplementary Material 5 [file 12873_2023_820_MOESM5_ESM.docx]

**Supplement 4:** Quality parameters of chest compressions

| **Parameter** | **ERC 2021**  **(n=30)** | | **COVID-19 -Intubation**  **(n=30)** | | **COVID-19-laryngeal mask**  **(n=30)** | | **COVID-19-shower-cap**  **(n=30)** | |
| --- | --- | --- | --- | --- | --- | --- | --- | --- |
|  | **Non-VF**  **(n=15)** | **VF**  **(n=15)** | **Non-VF**  **(n=15)** | **VF**  **(n=15)** | **Non-VF**  **(n=15)** | **VF**  **(n=15)** | **Non-VF**  **(n=15)** | **VF**  **(n=15)** |
| **Compression depth (mm)** | 50±5 | 47±6 | 44±7 | 50±5 | 43±5 | 49±5 | 45±6 | 50±5 |
| **Frequency of compressions (/min)** | 109±8 | 111±5 | 117±7 | 111±7 | 116±6 | 110±7 | 118±5 | 111±6 |
| **Compressions with** |  |  |  |  |  |  |  |  |
| - **correct pressure point (%)** | 95±10 | 95±10 | 98±3 | 86±20 | 99±4 | 90±14 | 96±15 | 81±28 |
| - **sufficient pressure depth (%)** | 54±37 | 41±32 | 22±30 | 42±32 | 18±21 | 42±35 | 28±30 | 48±33 |
| - **correct frequency (%)** | 77±24 | 82±19 | 64±29 | 80±26 | 67±30 | 82±25 | 62±31 | 83±25 |
| - **Complete relief (%)** | 55±38 | 72±32 | 84±17 | 70±29 | 81±15 | 70±25 | 73±27 | 68±29 |
| **Legend:** | COVID-19=Corona-Virus-Disease 2019; ERC=European Resuscitation Council; mg=milligram; mm=millimeters; min=minute; Non-VF=Non-shockable initial rhythm; sec=second, VF=Shockable initial rhythm  data are presented as mean ± standard deviation | | | | | | | |
